# Influence of Rearing Environment on Longitudinal Brain Development, Object Recognition Memory, and Exploratory Behaviors in the Domestic Pig (*Sus scrofa*)

**DOI:** 10.3389/fnins.2021.649536

**Published:** 2021-03-24

**Authors:** Joanne E. Fil, Sangyun Joung, Courtney A. Hayes, Ryan N. Dilger

**Affiliations:** ^1^Neuroscience Program, University of Illinois, Urbana, IL, United States; ^2^College of Veterinary Medicine, University of Illinois, Urbana, IL, United States; ^3^Department of Animal Sciences, University of Illinois, Urbana, IL, United States; ^4^Division of Nutritional Sciences, University of Illinois, Urbana, IL, United States

**Keywords:** pig, brain, neurodevelopment, behavior, magnetic resonance imaging, modeling, Gompertz, longitudinal

## Abstract

**Introduction:**

Over the last 40 years, the domestic pig has emerged as a prominent preclinical model as this species shares similarities with humans with regard to immunity, gastrointestinal physiology, and neurodevelopment. Artificial rearing of pigs provides a number of advantages over conventional rearing (i.e., true maternal care), including careful control of nutrient intake and environment conditions. Yet there remains a gap in knowledge when comparing brain development between sow-reared and artificially reared domestic pigs. Thus, our research sought to model brain development and assess recognition memory in a longitudinal manner by directly comparing rearing environments.

**Methods:**

Forty-four intact (i.e., not castrated) male pigs were artificially reared or sow-reared from postnatal day 2 until postnatal week 4. After postnatal week 4, all pigs were housed in a group setting within the same environment until postnatal week 24. Magnetic resonance imaging was conducted on pigs at 8 longitudinal time-points to model developmental trajectories of brain macrostructural and microstructural outcomes. Additionally, pigs behavior were tested using the novel object recognition task at postnatal weeks 4 and 8.

**Results:**

Throughout the 24-week study, no differences between rearing groups were noted in weekly body weights, average growth and feed intake patterns, or feed efficiency. Whole brain, gray matter, white matter, and cerebrospinal fluid growth patterns also did not differ between pigs assigned to different early-life rearing environments. Moreover, minimal differences in regional absolute volumes and fractional anisotropy developmental trajectories were identified, though artificially reared pigs exhibited higher initial rates of myelination in multiple brain regions compared with sow-reared pigs. Furthermore, behavioral assessment at both PNW 4 and 8 suggested little influence of rearing environment on recognition memory, however, an age-dependent increase in object recognition memory was observed in the sow-reared group.

**Conclusion:**

Our findings suggest that early-life rearing environment influences the rate of development in some brain regions but has little influence on overall brain growth and object recognition memory and exploratory behaviors in the domestic pig. Artificial rearing may promote maturation in certain brain areas but does not appear to elicit long-term effects in outcomes including brain structure or object recognition memory.

## Introduction

Preclinical animal models are crucial for addressing biological processes occurring in humans and provide an advantage in enabling invasive techniques to be used for investigating tissue development. The pig is a noteworthy preclinical model in a variety of fields, including those related to nutrition and development, as they resemble humans in morphology and functionality of multiple biological processes (Swindle et al., 1994; Sciascia et al., 2016; Mudd and Dilger, 2017). Similarly to humans, the pig brain develops perinatally and exhibits a peak growth rate around the time of birth (Dobbing and Sands, 1979; Pond et al., 2000). Additionally, macrostructure of the pig brain resembles human brain morphology in gyral patterning (Dickerson and Dobbing, 1967; Lind et al., 2007), brain regions (Saikali et al., 2010), and distribution of gray matter (GM) and white matter (WM) (Dickerson and Dobbing, 1967; Lind et al., 2007), thereby reinforcing the pig as an ideal model for neuroscience-related studies. Indeed, pigs have been used to model traumatic brain injury (Kinder et al., 2019), stroke (Wagner et al., 1999), and the influence of nutrition on neurodevelopment (Mudd et al., 2018; Fil et al., 2019).

Artificial rearing is a useful method to implement for interventions that require careful monitoring of the subject’s dietary intake or control of environment without sibling and maternal influence. This rearing environment involves raising animals in specialized enclosures that provide ready access to resources meeting nutritional, physiological, environmental, and social needs of the pig. By maintaining close control over the environment and contact with other subjects, researchers utilizing artificially reared (AR) animals can reduce variability within the research population, thereby providing greater statistical power in experiments. Moreover, the pig is an ideal animal model to artificially rear because they can be maintained starting at birth and exhibit growth rates comparable to sow-reared (SR) pigs (Braude et al., 1983; Cabrera et al., 2010). This is unlike rodents, which experience higher rates of mortality when reared artificially (Henare et al., 2008), potentially due to multiple factors, including any invasive surgery and the associated stress and anxiety experienced while in those environments (Yasuda et al., 2016).

Changes in social and physical environments induce stress in any species, including the pig, and studies have been conducted to evaluate whether artificial rearing influences pig development and behavior. Most studies focus on pig health and performance, as artificial rearing has become a strategy to manage large litters that are beyond the rearing capacity of a sow (Baxter et al., 2013). Behavioral analyses have revealed greater incidence of belly nosing (Rzezniczek et al., 2015; Schmitt et al., 2019) in AR pigs compared with SR pigs, in addition to other negative behaviors, some of which include aggression toward other pigs (Rzezniczek et al., 2015) and more frequent oral manipulation of conspecific tails and ears (Schmitt et al., 2019).

As pigs continue to be an important preclinical model for neuroscience-related research, we aimed to evaluate the influence of early-life rearing environment on longitudinal brain structural development and behavior. Our null hypothesis was that no differences in brain development would exist between AR and SR pigs. To test this hypothesis, the brain developmental patterns of absolute volume and myelination were evaluated longitudinally utilizing various magnetic resonance imaging (MRI) sequences and novel object recognition task was preformed to evaluate recognition memory.

## Materials and Methods

### Animals

Pregnant sows were obtained from a commercial swine farm (Carthage Veterinary Services, Carthage, IL) and transferred to the Veterinary Medicine Research Farm located at the University of Illinois at Urbana-Champaign 1 week prior to the farrowing (i.e., pig-specific term for giving birth). Sows were provided 15 mg of Matrix (Merck Animal Health/Intervet Inc., Madison, NJ) daily *per os* for 2–4 days prior to the expected farrowing date and injected with 0.7 mL of Estrumate (250 μg/ml; Merck Animal Health/Intervet Inc., Madison, NJ) one day before the expected farrowing date to synchronize farrowing events between sows. Once sows farrowed, pigs were provided with a single dose of prophylactic antibiotic [5.0 mg/kg body weight (BW); Excede, Zoetis, Kalamazoo, MI] and iron dextran (200 mg per pig; Uniferon 200, Pharmacosmos, Inc., Watchung, NJ) within the first 24 h of birth.

Pigs were allowed access to colostrum for up to 48 h and before being assigned to either SR or AR treatment groups, representing the two rearing environments tested in this study. From postnatal day (PND) 2 to 28, pigs either remained with their littermates and dam (i.e., the sow; SR group) or were transported to the Piglet Nutrition and Cognition Laboratory and artificially reared (AR group). Regardless of early-life rearing environment, all pigs received two doses of *Clostridium perfringens* antitoxins C and D (one 5 mL dose given subcutaneously and one 3 mL dose given orally; Colorado Serum Company, Denver, CO) as a prophylactic measure on PND 2 to avoid incidence of enterotoxemia that sometimes occurs in young pigs. The experiment was completed at postnatal week (PNW) 24 (i.e., approximate age of sexual maturity for pigs; Reiland, 1978) using 4 cohorts of pigs selected from 15 total litters (i.e., 3 or 4 sows per cohort) to control for initial BW and genetics for a total of 44 pigs completing the study. All animals and experimental procedures were conducted in accordance with the National Research Council Guide for the Care and Use of Laboratory Animals and approved by the University of Illinois at Urbana-Champaign Institutional Animal Care and Use Committee.

### Housing

**FIGURE 1:**
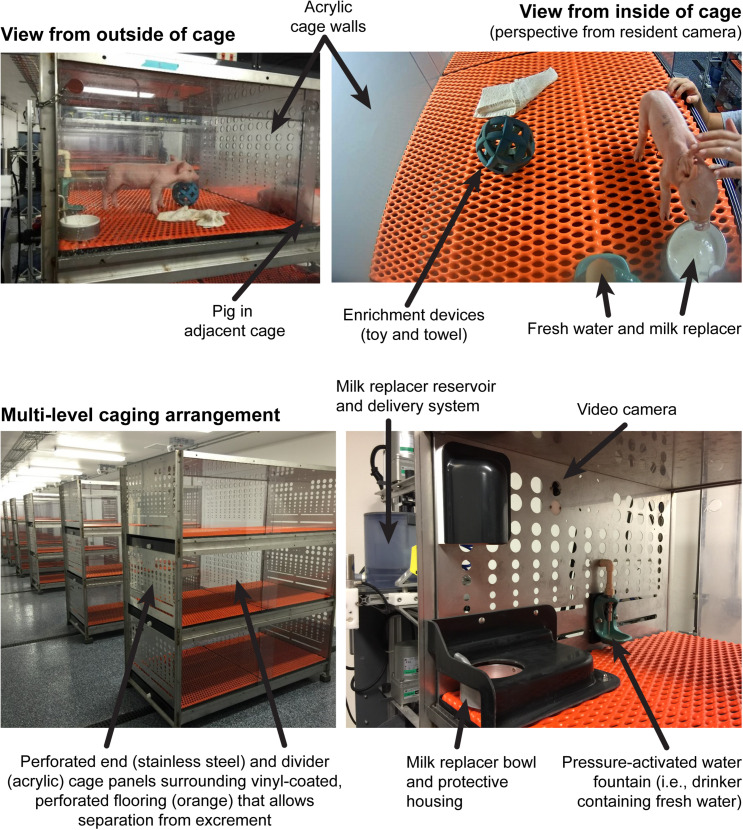
Visual description of the artificial rearing (AR) environment. This context includes individual pigs each housed in a dedicated cage with *ad libitum* access to fresh water and milk replacer and no competition for resources. Pigs are able to see, hear, and smell, but not directly touch, neighboring pigs through the clear, acrylic divider between cages on the same vertical level, as well as other pigs in adjacent caging units. Whereas, young pigs have difficulty with thermoregulation due to low body fat stores, the ambient AR environment is maintained at optimal temperatures and relatively humidity levels for each age of pig. Pigs raised in the AR environment are handled by human caretakers at least twice daily, which is why AR pigs appear less anxious relative to SR pigs when exposed to experimental procedures. Whereas, AR pigs are technically raised in their own space, their ability to engage with other pigs both in their home-cage and during daily communal activity periods mean they are not socially isolated and still receive attention from caregivers in the absence of natural maternal care.

Through PNW 4, AR pigs were housed individually in custom rearing units (87.6 cm long, 88.9 cm wide, 50.8 cm high), which were composed of three acrylic walls, one stainless steel wall, and vinyl-coated, expanded-metal flooring. This caging environment allowed pigs to see, hear, and smell, but not touch, neighboring pigs (Figure 1). Each pig was provided a toy for enrichment in their home-cage and were allowed to physically interact with one another for approximately 15 min each day. Lights were automatically controlled (12-h cycle, on from 0800 h to 2000 h) with ambient temperature set at 26.6°C for the first 21 days of the study and gradually lowered to 22°C during the last 7 days of housing at the Piglet Nutrition and Cognition Laboratory.

**FIGURE 2:**
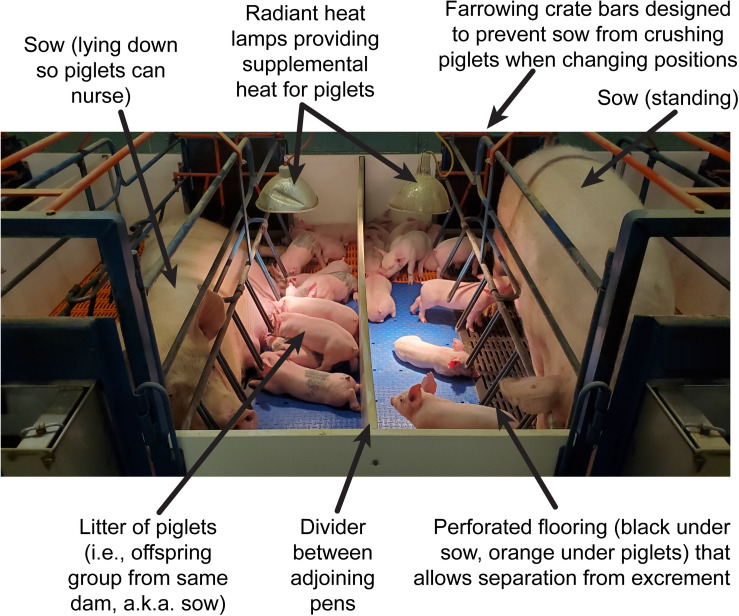
Visual description of the sow-reared (SR) environment. This context includes an individual dam (i.e., sow) housed within a farrowing (i.e., porcine-specific term for giving birth) crate that is designed to prevent the sow from crushing piglets when changing to a lying position. Within the outer area of the pen, all pigs within a litter (i.e., group of piglets all born to the same dam) engage directly with each other and with the dam, including competition between littermates for access to teats when obtaining milk as the sole source of nutrition, including water. Pigs in this rearing environment are also exposed to excrement and bodily fluids from both the dam and littermates, which has implications on development of the microbiota. In this context, it is impossible to discern how a dietary intervention will individual piglets as there is no control over access to milk availability or a way for development of heterogeneous microbiota. Additionally, the phenotypic behavioral traits of SR pigs include a clear tendency to run away from human caregivers (i.e., relatively high apparent anxiety compared with artificially reared pigs) as there is limited human interaction on a routine basis.

All SR pigs remained with their respective dam and littermates in farrowing crates at the Veterinary Medicine Research Farm through PNW 4, per standard agricultural practices (Figure 2). This rearing environment (i.e., a university-owned swine facility) was maintained at a constant 22°C with a light cycle identical to that of AR pigs (12-h cycle, on from 0800 h to 2000 h). Daily health assessments of all AR and SR pigs were recorded through PNW 4 to track incidence of diarrhea, lethargy, weight loss, or vomiting as clinical indicators. After PNW 4, AR pigs were transported back to the originating farm (Veterinary Medicine Research Farm) and were group-housed with SR pigs (3–4 pigs per pen) until PNW 8 in age-appropriate, raised deck pens (1.219 m × 1.219 m; vinyl-coated, expanded metal flooring, with one nipple drinker and 4-hole feeder per pen; *ad libitum* access to feed). After PNW 8, all pigs were moved to larger pens (1.676 m × 3.658 m; solid concrete floors with one nipple drinker and feed provided twice-daily per pen (feed amount based on pig age and pen group weight), with pigs remaining in the same group as beginning at PNW 4.

### Feeding Procedures

All AR pigs (*n* = 31) received a custom bovine milk-based milk replacer formula (TestDiet, Richmond, IN) formulated to meet all nutritional requirements for the young pig (National Research Council, 2012) from PND 2 to PNW 4 (see Table 1 for nutrient composition of milk replacer). As such, AR pigs had *ad libitum* access to liquid milk replacer using an automated delivery system that dispensed milk from 1000 h to 0600 h the next day (20-h daily feeding cycle). Milk replacer was reconstituted fresh daily at 200 g of dry powder per 800 g of tap water. Daily weights of individual pigs and their respective milk reservoirs were recorded. The remaining volume of milk was subtracted from the initial volume provided to quantify milk disappearance over the 20-h feeding period, which will henceforth be referred to as milk intake. SR pigs (*n* = 13) only had access to maternal milk (i.e., no other sources of nutrition were accessible) through PNW 4. Once all animals were transitioned to group-housing at the Veterinary Medicine Research Farm after PNW 4, both AR and SR pigs were maintained on a common series of industry-standard, nutritionally adequate diets through PNW 24 (i.e., study conclusion). Thus, the only difference between AR and SR pigs was that the rearing environment (including diet) differed from PND 2 to PNW 4, with all factors being equalized thereafter.

**TABLE 1:** Nutrient composition of milk replacer.

Nutrient	Value
**Energy and macronutrients**	
Energy, kcal/g	4.26
Carbohydrate, %	34.30
Fat, %	39.10
Protein, %	26.60
**Minerals**	
Ash, %	6.50
Calcium, %	1.68
Phosphorus, %	0.78
Phosphorus (available), %	0.76
Potassium, %	0.95
Magnesium, %	0.09
Sulfur, %	0.24
Sodium, %	0.76
Chloride, %	1.00
Fluorine, ppm	12.30
Iron, ppm	142.00
Zinc, ppm	103.00
Manganese, ppm	47.00
Copper, ppm	19.00
Cobalt, ppm	0.62
Iodine, ppm	1.18
Chromium (added), ppm	0.02
Selenium, ppm	0.30
**Vitamins**	
Vitamin A, IU/g	3.00
Vitamin D-2 (added), IU/g	6.70
Vitamin E, IU/kg	330.00
Vitamin K, ppm	5.00
Thiamin, ppm	3.00
Riboflavin, ppm	13.20
Niacin, ppm	60.00
Pantothenic acid, ppm	30.00
Folic acid, ppm	0.90
Pyridoxine, ppm	3.00
Biotin, ppm	0.30
Vitamin B-12, mcg/kg	110.00
Choline chloride, ppm	1700.00
Ascorbic acid, ppm	49.20

### Magnetic Resonance Imaging

Pigs underwent MRI procedures at 1, 2, 3, 4, 8, 12, 18, and 24 weeks of age at the Beckman Institute for Advanced Science and Technology (University of Illinois, Urbana, IL) Biomedical Imaging Center using a MAGNETOM Prisma 3T MRI scanner (Siemens; Munich, Germany). A custom 8-channel head coil designed for young pigs was used through PNW 4 (Rapid Biomedical; Rimpar, Germany) and 32-channel spine and 18-channel flex coils (Siemens; Munich, Germany) were used for scans occurring from PNW 8–24. Upon arrival to the imaging facility, pigs were anesthetized using a combination of telazol:ketamine:xylazine [50.0 mg tiletamine plus 50.0 mg of zolazepam reconstituted with 2.50 mL ketamine (100 g/L) and 2.50 mL xylazine (100 g/L); Fort Dodge Animal Health, Overland Park, KS] by i.m. injection at 0.03 mL/kg BW. Once anesthetized, pigs were placed in a supine position in the MRI machine and kept under sedation by inhalation of isoflurane (0.6–2.0% using a progressive dosing regimen based on pig BW) with the balance as pure oxygen throughout the entire procedure (total scan time was approximately 75-min per session).

Oxygen saturation levels and heart rate were monitored using two pulse oximeters (LifeWindow LW9x, Boynton Beach, FL and MEDRAD Veris 8600, Indianola, PA) each with an infrared sensor that was clipped on the pig’s tail and/or left-hind hoof. Observational records of heart rate, partial pressure of oxygen, and percent of isoflurane were recorded every 5 min after anesthetic induction. The pig neuroimaging protocol included a magnetization prepared rapid gradient-echo sequence and diffusion tensor imaging (DTI) to assess brain macrostructure and microstructure, respectively. The multicomponent driven equilibrium single pulse observation of T_1_ and T_2_ technique was used to measure myelin-associated water fraction (MWF). Imaging techniques are described in greater detail below.

#### Structural MRI Acquisition and Analysis

A T1-weighted a magnetization prepared rapid gradient-echo sequence was used to obtain anatomic images of the pig brain throughout the 24-wk study. The following sequence-specific parameters were used to acquire T1-weighted a magnetization prepared rapid gradient-echo data through PNW 4: repetition time = 2000.0 ms; echo time = 2.05 ms; inversion time = 1,060 ms, flip angle = 9°, matrix = 288; slice thickness = 0.6 mm. Parameters for pigs from PNW 8–24 were as follows: repetition time = 2060.0 ms; echo time = 1.71 ms; inversion time = 1,060 ms, flip angle = 9°, matrix = 256; slice thickness = 1.0 mm The final voxel size was 0.6 mm isotropic across the entire head from the tip of the snout to the cervical/thoracic spinal cord junction in pigs through PNW 4, and was 1.0 mm isotropic in pigs from PNW 8–24. Detailed image processing and volume estimation methods has been previously described (Fil et al., 2021).

#### Myelin Water Fraction Acquisition and Analysis

The multicomponent driven equilibrium single pulse observation of T_1_ and T_2_ technique was used to measure myelin-associated water fraction throughout the brain of pigs to provide insight into brain myelination patterns. For pigs 4 weeks and younger, a constant 7.0 × 10.8 × 14.7 mm^3^ sagittally oriented field of view with 160 × 160 × 125 imaging matrix was used, providing a voxel volume of 1.7 × 1.7 × 2.6 mm^3^. The spoiled gradient echo and T_2_/T_1_ – weighted balanced steady-state free precession (SSFP) data were acquired with the following sequence-specific parameters: spoiled gradient echo, echo time (TE)/repetition time (TR) = 2.7 ms/5.6 ms; receiver bandwidth = 350 Hz/voxel; and SSFP, TE/TR flip angles = 2.6 ms/5.3 ms; receiver bandwidth = 350 Hz/voxel. For pigs older than 4 weeks, a constant 4.1 × 5.4 × 31.5 mm^3^ sagittally oriented field of view with 260 × 260 × 240 imaging matrix was used, providing a voxel volume of 2.7 × 2.7 × 3.0 mm^3^. The spoiled gradient echo and SSFP data were acquired with the following sequence-specific parameters: spoiled gradient echo, TE/TR = 2.7 ms/5.6 ms; receiver bandwidth = 350 Hz/voxel; and SSFP, TE/TR = 2.6 ms/5.3 ms; receiver bandwidth = 350 Hz/voxel. Two sets of SSFP data were acquired with phase-cycling increments of both 0° and 180° to allow for correction of main magnetic field (i.e., off-resonance) artifacts. Processing of MWF data was performed using methods described previously (Deoni et al., 2011) with modifications to the sequence only including a change in the threshold used for imaging data collected for the pig.

#### Diffusion Tensor Imaging Acquisition and Analysis

DTI was used to assess WM maturation and axonal tract integrity using a diffusion-weighted echo planar imaging sequence with the following parameters for pigs aged 4 weeks and younger: repetition time = 5,100 ms; echo time = 70 ms; generalized auto-calibrating partially parallel acquisitions accelerated by a factor of 2 in the phase encode direction; diffusion weightings = 1,000 and 2,000 s/mm^2^ across 30 directions; 1 image with a b-value of 0 s/mm^2^. Fifty slices with a 1.6 mm thickness were collected with a matrix size of 100 × 100 for a final voxel size of 1.6 mm isotropic. In pigs older than 4 weeks, the parameters were as follows: repetition time = 5,600 ms; echo time = 70 ms; generalized auto-calibrating partially parallel acquisitions accelerated by a factor of 2 in the phase encode direction; diffusion weightings = 1,000 and 2,000 s/mm^2^ across 30 directions; 1 image with a b-value of 0 s/mm^2^. Fifty-four slices with a 2.0 mm thickness was collected with a matrix size of 130 × 130 for a final voxel size of 2.0 mm isotropic.

Diffusion-weighted echo planar imaging images were assessed in the FMRIB Software library (Jenkinson et al., 2012) to generate values of fractional anisotropy (FA) using methods previously described (Fil et al., 2021). Assessment was performed over the following regions of interest: both caudate nuclei, corpus callosum, cerebellum, both hippocampi, both internal capsules, left and right sides of the brain, thalamus, DTI-generated WM, and atlas-generated WM. This assessment was performed using a customized analysis pipeline designed for the pig and the FMRIB Software library package described in Fil et al. (2021). In the corresponding results, atlas-generated WM indicated the use of WM prior probability maps from the pig brain atlas that were used as a region of interest mask. Likewise, DTI-generated WM indicated a threshold of 0.2 should be applied to FA values, thus restricting analysis to WM tracts only.

### Behavioral Testing

Novel object recognition (NOR), described in detail previously (Fleming and Dilger, 2017), was used to assess recognition memory as a primary indicator of cognitive behavior of the pig. Testing consisted of a habituation phase, a sample phase, and a test phase. During the habituation phase, each pig was placed in an empty testing arena for 10 min for 2 days leading up to the sample phase. In the sample phase, two identical objects were placed in the arena and pigs were given 5 min for exploration. After a delay of 48 h, the test phase as conducted where pigs were returned to the arena, which contained one object from the sample phase as well as a novel object. Between trials, objects were removed, immersed in hot water with detergent, and rubbed with a towel to mitigate odor while the arena was sprayed with water to remove urine and feces. Objects chosen had a range of characteristics (i.e., color, texture, shape, and size), however, the novel and sample objects only differed in shape and size. Only objects previously shown to elicit a null preference were used for testing. The NOR task was completed at two different time-points, PNW 4 and 8. At PNW 4, the habituation trial began at PND 24, and the testing trial at PND 28. At PNW 8, habituation trials began at PND 52, and the testing trial began at PND 56. However, not all pigs that completed the NOR task at PNW 4 were able to complete the task at PNW 8, due to failure to thrive. The object set used for each time-point was different and counterbalanced. Recognition index, or the proportion of time spent investigating the novel object compared with the total exploration time of both objects, was compared to a chance performance value of 0.50 to assess recognition memory. Values greater than 0.50 were interpreted as being indicative of a novelty preference, thus suggesting pigs exhibited recognition memory.

### Statistical Analysis

All statistical models included replicate and litter as random effects and the level of significance was set at *P* < 0.05. Growth and milk intake data for each individual pig was subjected to an Analysis of Variance using SAS 9.3 (SAS Inst. Inc., Cary, NC). Data for growth and milk intake was analyzed as a repeated-measures Analysis of Variance also using the MIXED procedure, and exploratory behavior was analyzed as a two-way Analysis of Variance with the age and rearing system as main effects. To assess recognition memory, the recognition index was compared to a chance performance value of 0.50 using a one-sample *t*-test.

#### Brain Macrostructure and Microstructure Modeling

**FIGURE 3:**
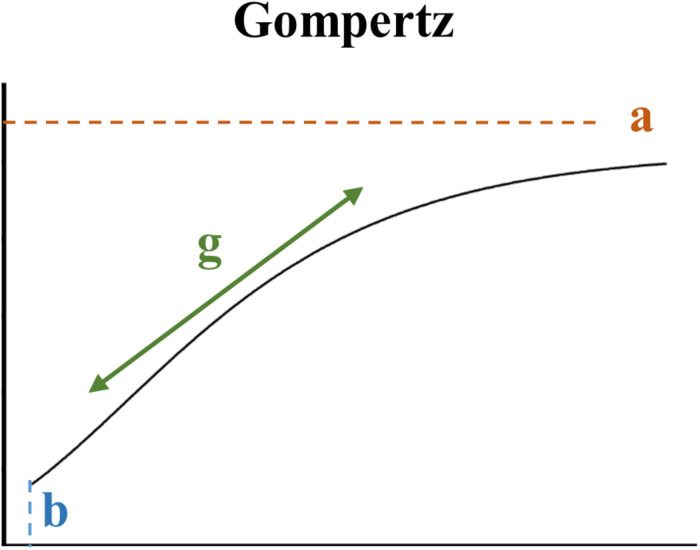
Properties of the Gompertz model. Parameters indicate maximum (i.e., plateau) value (a), age at initial onset of tissue development (b), and overall rate of tissue development (g).

Absolute volume, FA, and MWF developmental models were constructed for each brain area in each pig using the NLMIXED method in SAS 9.3. Parameter estimations were computed for nine different sigmoid-type models. The growth models of Gompertz (Gompertz, 1825), Bleasdale and Nelder (Bleasdale and Nelder, 1960), Richards (Richards, 1959), Stannard (Stannard et al., 1985), a modified Gompertz from Dean et al. (Dean et al., 2014), two different logistic functions (Conrad et al., 2012; Dean et al., 2014), the generalized logistic, and the hyperbolic tangent were all fitted. The Bayesian information criterion (BIC) was measured for each model in each ROI. The BIC is a criterion for model selection where the lowest BIC is preferred because unexplained variation in the dependent variable and the number of explanatory variables increase the value of BIC (Schwarz, 1978). The Gompertz (Figure 3) was chosen as the best model to use for absolute volume, MWF, and FA data because it had the best BIC value across all brain regions using the highest ranked sum (Supplementary Tables 1–3).

The Gompertz model was parameterized as follows:

O⁢u⁢t⁢c⁢o⁢m⁢e=a*exp⁢(-1*exp⁡(b-g*P⁢N⁢W))

where the outcome was either absolute volume, MWF, or FA, and PNW indicated postnatal week (i.e., age of the pig). Parameter estimations for each outcome were computed for maximum absolute (i.e., plateau) value (a), onset of initial developmental increase (b), and overall rate of development (g). A two-sample *t*-test was conducted in SAS 9.3 to compare individual modeled parameter estimates between SR and AR pigs.

## Results

### Growth Performance and Feed Intake

#### Body Weight and Feed Intake

**FIGURE 4:**
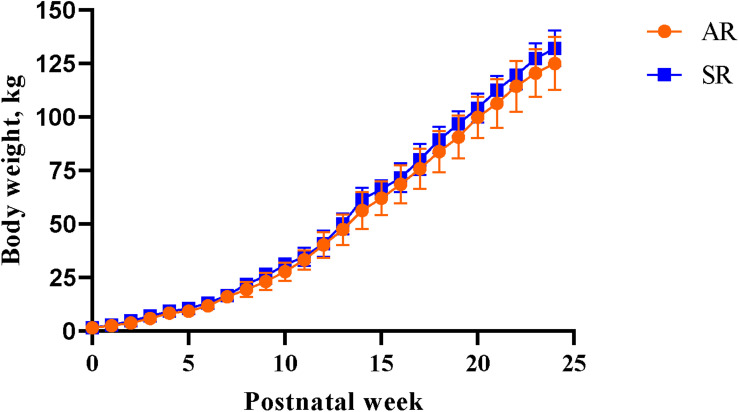
Weekly body weights of artificially reared pigs and sow-reared pigs from postnatal day 2 until postnatal week 24. AR, artificially reared pigs; BW, body weight; PNW, postnatal week; SR, sow-reared pigs.

A main effect of PND was observed for daily BW (*P* < 0.001), meaning that there was an active growth phase over the study period. There was no main effect of rearing environment on BW (*P* > 0.05) indicating that all pigs gained a similar amount of weight over time, regardless of the rearing environment (Figure 4). A main effect of PNW on feed intake was observed, where pigs consumed more feed (*P* < 0.001) as the study progressed. Regardless of early-life rearing environment, all pigs consumed the same amount of feed (*P* > 0.05) over the latter part of the study.

#### Growth and Feed Performance

Effects of rearing environment on average daily gain, average daily feed intake, and gain-to-feed ratio are presented in Table 2. No differences between rearing environments were observed for any measure.

**TABLE 2:** Growth and feeding performance on milk replacer (PND 3–28) and feed (PNW 5–24)^*a*^.

	Diet		Pooled	
Measure	AR	SR	SEM	*P*-value^*b*^
**PNW 1–4**				
ADG, kg/day	0.257	0.276	0.015	0.167
ADFI, kg liquid milk replacer/day	1.377	–	–	–
G:F, kg BW:kg liquid milk replacer	0.187	–	–	–
**PNW 5–24**				
ADG, kg/day	0.811	0.913	0.059	0.153
ADFI, kg solids/day	4.323	4.492	0.187	0.368
G:F, kg BW:kg solids	0.175	0.204	0.016	0.127

*^*a*^ADG, average daily gain; ADFI, average daily feed intake; AR, artificially reared pigs; BW, body weight; G:F, gain to feed; PND, postnatal day; PNW, postnatal week; SEM, standard error of the mean; SR, sow-reared pigs.*

*^*b*^P-values derived from a repeated-measures mixed model ANOVA comparing pigs raised in different early-life rearing environments.*

### Brain Analysis

#### Absolute Volumes

**FIGURE 5:**
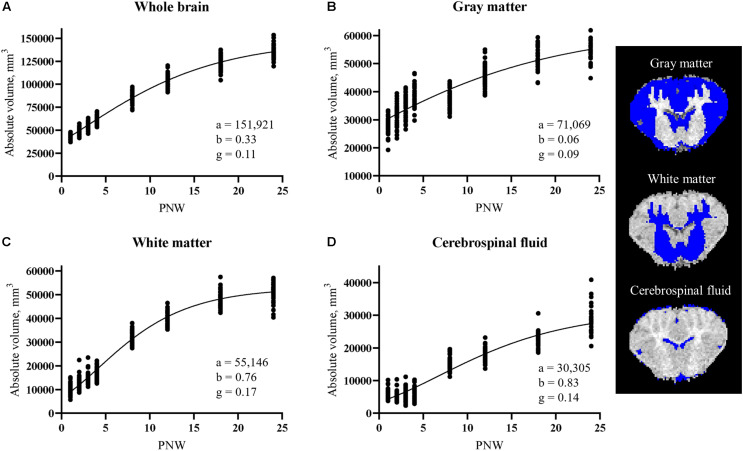
Developmental patterns of absolute volume in **(A)** whole brain, **(B)** gray matter, **(C)** white matter, and **(D)** cerebrospinal fluid in pigs. Parameters indicate maximum (i.e., plateau) value (a), age at initial onset of tissue development (b), and overall rate of tissue development (g). Rearing environment did not influence developmental patterns, therefore data from artificially reared pigs and sow-reared pigs were combined. Coronal brain slices displaying gray matter, white matter, and cerebrospinal fluid tissue in blue. PNW, postnatal week.

Most absolute volume parameter estimates were not different due to rearing environment, including whole brain, GM, WM, and cerebrospinal fluid volumes (Figure 5). The only significant differences observed was a higher (*P* < 0.05) overall rate of development in the left olfactory bulb and right and left cortex of SR pigs than AR pigs. Parameter estimates for all regions can be found in Table 3.

**TABLE 3:** Brain region-specific parameter estimates for absolute volumes generated using the Gompertz model^a^.

	AR			SR			Pooled SEM			*P*-value^b^		
ROI Parameter^c^	a	b	g	a	b	g	a	b	g	a	b	g
Whole brain	151089.61	0.34	0.11	153903.08	0.32	0.11	4997.735	0.015	0.007	0.576	0.059	0.667
Gray matter	69604.52	0.08	0.11	72534.30	0.04	0.07	8589.139	0.180	0.045	0.733	0.805	0.315
White matter	53922.68	0.77	0.17	56369.77	0.74	0.17	1392.552	0.033	0.009	0.116	0.355	0.985
Cerebrospinal fluid	30667.48	0.86	0.13	29942.62	0.80	0.14	2884.166	0.040	0.015	0.782	0.132	0.308
Cerebral aqueduct	80.68	0.03	0.10	73.15	–0.09	0.08	12.047	0.135	0.051	0.536	0.380	0.479
Corpus callosum	1505.90	0.42	0.08	1625.90	0.41	0.10	333.400	0.068	0.016	0.721	0.906	0.226
Cerebellum	26643.50	0.47	0.09	23953.70	0.51	0.10	6700.800	0.092	0.018	0.690	0.651	0.626
Fourth ventricle	211.60	–0.05	0.06	176.80	–0.05	0.07	42.549	0.166	0.009	0.279	0.986	0.245
Hypothalamus	389.00	0.36	0.10	434.80	0.43	0.11	42.842	0.040	0.013	0.292	0.106	0.491
Left caudate	1095.20	0.27	0.06	1263.80	0.28	0.08	258.000	0.105	0.013	0.599	0.939	0.421
Left cortex	45716.40	0.40	0.10	45043.70	0.37	0.14	2781.800	0.116	0.013	0.764	0.704	**0.013**
Left hippocampus	1458.70	0.44	0.08	1315.20	0.43	0.10	227.700	0.060	0.014	0.439	0.843	0.279
Left inferior colliculus	382.30	0.48	0.07	424.20	0.46	0.08	59.245	0.091	0.014	0.484	0.796	0.420
Left internal capsule	2246.40	0.33	0.08	2336.10	1.30	–0.01	414.200	0.641	0.078	0.868	0.349	0.471
Left olfactory bulb	3805.20	0.43	0.11	3721.50	0.43	0.14	388.700	0.097	0.015	0.775	0.980	**0.011**
Left putamen-globus pallidus	759.80	0.33	0.06	679.10	0.31	0.08	154.400	0.106	0.015	0.604	0.839	0.189
Left superior colliculus	898.20	0.46	0.07	941.30	0.45	0.08	163.100	0.080	0.013	0.793	0.975	0.276
Lateral ventricle	2030.70	0.36	0.07	2088.50	2.73	2.06	409.400	1.531	1.256	0.888	0.343	0.333
Medulla	6267.00	0.63	0.09	5610.70	0.72	0.10	1324.200	0.136	0.017	0.476	0.620	0.584
Midbrain	6118.80	0.34	0.09	6499.20	0.37	0.10	258.400	0.038	0.009	0.149	0.471	0.711
Pons	4101.10	0.51	0.09	5598.90	0.49	0.11	991.600	0.098	0.013	0.322	0.824	0.153
Right caudate	1485.60	0.38	0.06	1159.10	0.28	0.07	424.000	0.111	0.015	0.318	0.384	0.384
Right cortex	44509.30	0.40	0.10	45076.90	0.35	0.13	2725.700	0.118	0.013	0.836	0.481	**0.019**
Right hippocampus	1372.30	0.50	0.08	1458.40	0.41	0.09	281.000	0.114	0.013	0.761	0.343	0.191
Right inferior colliculus	476.20	0.53	0.06	491.50	0.45	0.08	95.504	0.093	0.015	0.902	0.357	0.165
Right internal capsule	2107.90	0.32	0.08	1981.60	0.26	0.10	234.100	0.056	0.013	0.593	0.281	0.086
Right olfactory bulb	3663.90	0.37	0.13	3755.30	0.45	0.13	499.800	0.100	0.065	0.856	0.468	0.978
Right putamen-globus pallidus	576.30	0.26	0.07	535.10	0.26	0.09	74.025	0.091	0.012	0.581	0.97	0.138
Right superior colliculus	840.50	0.41	0.08	788.50	0.42	0.08	143.500	0.083	0.015	0.719	0.899	0.772
Thalamus	3474.80	0.33	0.06	3631.40	0.36	0.06	347.000	0.145	0.010	0.655	0.833	0.749

*^a^AR, artificially reared pigs; ROI, region of interest; SEM, standard error of the mean; SR, sow-reared pigs.*

*^b^P-values derived from two-sample t-test comparing pigs raised in different early-life environments and group-housed in a common setting starting at postnatal week 4. Significant P-values are shown in bold text for emphasis.*

*^c^Parameter estimations for each outcome were computed for maximum absolute (i.e., plateau) value (a), onset of initial developmental increase (b), and overall rate of development (g).*

#### Myelin Water Fraction

**FIGURE 6:**
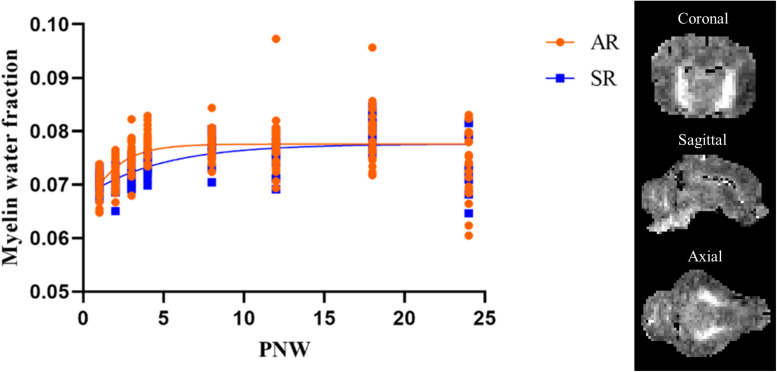
Myelin water fraction developmental pattern for the whole brain in artificially reared and sow-reared pigs. A distinct increase in myelin water fraction is evident during PNW 1–4 indicating a high period of myelination during early-life. Artificially reared pigs exhibited higher rates of myelin water fraction development than sow-reared pigs, however, that did not influence long-term development as plateau myelin water fraction estimates were similar between both groups. Myelin water fraction brain slices from one pig are presented in three different orthogonal views. AR, artificially reared pigs; PNW, postnatal week; SR, sow-reared pigs.

Rearing environment did not influence maximum mean MWF for pigs in either rearing environment, but the onset of MWF in the pons occurred earlier (*P* < 0.05) in SR pigs than AR pigs. AR pigs had higher (*P* < 0.05) overall rate of MWF development in the cerebellum, combined cortex, left internal capsule, left putamen-globus pallidus, midbrain, pons, right cortex, right hemisphere, thalamus, and whole brain. Figure 6 presents the slight variation in the developmental curve of the whole brain when overall rate of MWF development differs. The parameter estimates for all regions are presented in Table 4.

**TABLE 4:** Brain region-specific parameter estimates for myelin water fraction generated using the Gompertz model^a^.

	AR			SR			Pooled SEM			*P*-value^b^		
ROI Parameter^c^	a	b	g	a	b	g	a	b	g	a	b	g
Corpus callosum	0.24	–0.36	0.29	0.12	–0.57	0.26	0.107	0.281	0.099	0.072	0.280	0.827
Cerebellum	2.64	5.97	2.77	0.16	–3.85	1.04	4.097	12.513	1.186	0.333	0.222	**0.039**
Combined cortex	0.17	–1.02	0.31	0.12	–0.98	0.15	0.116	0.269	0.056	0.523	0.892	**0.001**
Combined hippocampus	0.10	–0.73	0.33	0.12	–0.67	0.27	0.033	0.147	0.056	0.554	0.711	0.346
Combined internal capsule	0.57	–0.35	0.28	0.36	–0.42	0.16	0.391	0.331	0.115	0.458	0.842	0.173
Hypothalamus	2.04	1.98	0.46	0.21	–0.45	0.54	3.031	3.018	0.324	0.334	0.205	0.849
Left cortex	0.21	–0.57	0.34	0.10	–0.86	0.13	0.144	0.811	0.169	0.206	0.573	0.062
Left hemisphere	0.12	–6.06	1.27	0.09	–1.34	0.15	0.040	8.875	1.250	0.245	0.393	0.159
Left hippocampus	0.31	–0.30	0.30	0.16	–0.72	0.27	0.173	0.332	0.095	0.212	0.110	0.623
Left inferior colliculus	2.15	14.72	8.01	0.14	–2.83	1.28	3.136	25.488	7.454	0.306	0.272	0.153
Left internal capsule	0.26	–0.28	0.41	0.60	–0.03	0.13	0.160	0.380	0.178	0.109	0.436	**0.024**
Left olfactory bulb	0.10	–3.93	8.93	0.09	5.34	5.95	0.057	6.651	3.883	0.853	0.254	0.447
Left putamen globus-pallidus	2.77	6.60	0.32	0.49	–0.01	0.13	4.082	11.219	0.122	0.369	0.344	**0.028**
Left superior colliculus	0.19	15.3	1.49	0.10	–0.67	0.59	0.098	24.494	0.908	0.108	0.296	0.134
Medulla	2.41	14.97	7.56	0.09	–1.86	5.40	3.790	29.193	1.734	0.326	0.357	0.118
Midbrain	0.13	–0.01	1.10	0.20	–1.00	0.44	0.063	1.017	0.399	0.462	0.145	**0.024**
Pons	2.69	15.90	12.12	0.08	–16.41	3.03	3.614	24.413	4.958	0.250	**0.040**	**0.008**
Right cortex	0.07	–4.12	0.35	0.14	–0.93	0.17	0.051	4.924	0.058	0.260	0.300	**0.004**
Right hemisphere	0.09	–1.19	0.53	0.21	–1.04	0.23	0.058	0.244	0.110	0.222	0.619	**0.008**
Right hippo	0.10	–0.76	0.32	0.09	–0.74	0.30	0.021	0.116	0.052	0.624	0.816	0.633
Right inferior colliculus	0.17	0.19	2.88	0.12	1.77	3.09	0.096	3.336	1.090	0.379	0.460	0.846
Right internal capsule	0.42	–0.19	0.20	0.34	–0.64	0.26	0.176	0.270	0.087	0.663	0.105	0.485
Right olfactory bulb	0.10	–2.62	6.53	0.10	101.90	4.89	0.048	60.775	2.337	0.899	0.317	0.486
Right putamen globus-pallidus	0.41	–0.10	0.21	0.24	–0.47	0.14	0.151	0.277	0.089	0.159	0.190	0.312
Right superior colliculus	0.11	–0.41	0.59	0.94	–0.48	0.33	0.502	0.392	0.179	0.333	0.832	0.054
Thalamus	0.19	–0.64	0.25	0.15	–0.62	0.14	0.100	0.186	0.054	0.594	0.930	**0.012**
Whole brain	0.13	–0.39	0.56	0.15	–2.55	0.10	0.065	2.257	0.167	0.852	0.343	**0.001**

*^a^AR, artificially reared pigs; ROI, region of interest; SEM, standard error of the mean; SR, sow-reared pigs.*

*^b^P-values derived from two-sample t-test comparing pigs raised in different early-life environments and group-housed in a common setting starting at postnatal week 4. Significant P-values are shown in bold text for emphasis.*

*^c^Parameter estimations for each outcome were computed for maximum absolute (i.e., plateau) value (a), onset of initial developmental increase (b), and overall rate of development (g).*

#### Fractional Anisotropy

All pigs had similar estimates for maximum FA values and onset of initial developmental increase, but SR pigs had a higher overall rate of increase of FA in the right internal capsule compared with AR pigs (*P* < 0.05). Parameter estimates for all regions can be found in Table 5.

**TABLE 5:** Brain region-specific parameter estimates for fractional anisotropy generated using the Gompertz model^a^.

	AR			SR			Pooled SEM			*P*-value^b^		
ROI parameter^c^	a	b	g	a	b	g	a	b	g	a	b	g
Corpus callosum	0.30	−1.00	1.00	0.30	−1.00	1.00	0.001	0.002	0.001	0.449	0.405	0.748
Cerebellum	0.31	−1.00	1.00	0.31	−1.00	1.00	0.002	0.001	0.001	0.167	0.557	0.745
Left caudate	0.31	−1.00	1.00	0.30	−1.00	1.00	0.002	0.000	0.000	0.858	0.340	0.584
Left hippocampus	0.36	−1.08	1.04	0.37	−1.13	1.12	0.005	0.040	0.042	0.213	0.181	0.160
Left internal capsule	0.31	−1.00	1.00	0.32	−1.01	1.01	0.005	0.001	0.001	0.513	0.405	0.326
Left side	0.52	−1.61	1.75	0.53	−1.37	2.09	0.008	0.294	0.263	0.050	0.287	0.106
Right caudate	0.34	−1.00	1.00	0.35	−1.00	1.00	0.006	0.001	0.002	0.799	0.139	0.355
Right hippocampus	0.30	−1.00	1.00	0.30	−1.00	1.00	0.002	0.000	0.000	0.235	0.093	0.093
Right internal capsule	0.32	−1.00	1.00	0.33	−1.00	1.01	0.008	0.001	0.002	0.081	0.687	**0.013**
Right side	0.51	−1.52	1.51	0.53	−1.35	2.23	0.008	0.377	0.289	0.090	0.374	0.639
Thalamus	0.37	−1.06	0.98	0.38	−1.04	0.99	0.005	0.024	0.020	0.253	0.150	0.142
FA mask	0.31	−1.00	0.99	0.32	−1.01	1.01	0.003	0.011	0.012	0.052	0.353	0.347
White matter mask	0.37	−1.16	1.12	0.37	−1.23	1.24	0.003	0.069	0.065	0.743	0.266	0.061

*^a^AR, artificially reared pigs; ROI, region of interest; SEM, standard error of the mean; SR, sow-reared pigs.*

*^b^P-values derived from two-sample t-test comparing pigs raised in different early-life environments and group-housed in a common setting starting at postnatal week 4. Significant P-values are shown in bold text for emphasis.*

*^c^Parameter estimations for each outcome were computed for maximum absolute (i.e., plateau) value (a), onset of initial developmental increase (b), and overall rate of development (g).*

### Behavioral Outcomes

**FIGURE 7:**
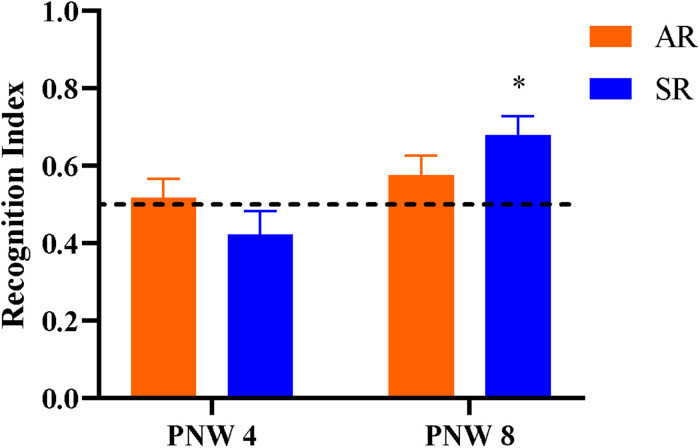
Recognition index during the test trial of the novel object recognition task as a measure of recognition memory. The dotted line at 0.50 indicates the chance performance value, and the recognition index greater than 0.50 indicates a novelty preference, and thus recognition memory. Note that an asterisk (*) on the PNW 8 sow-reared group denotes recognition memory as the recognition index was greater (*P* = 0.003) than the chance level (0.50 value). Other groups lacking an asterisk did not exhibit recognition memory (*P* > 0.05). AR, artificially reared pigs; PNW, postnatal week; SR, sow-reared pigs.

**FIGURE 8:**
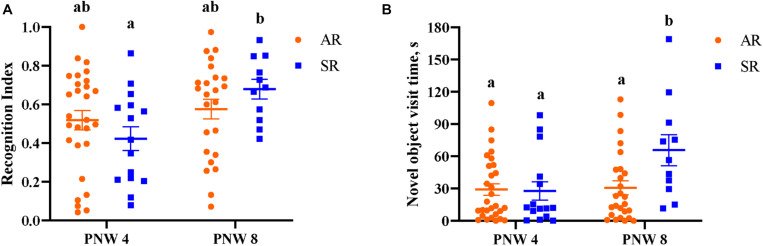
**(A)** Sow-reared pigs at PNW 4 and PNW 8 displayed difference in recognition index. Eight-week-old sow-reared pigs exhibited higher recognition index (*P* = 0.01) than 4-week-old sow-reared pigs, and similar effect of age was not evident in artificially reared pigs. **(B)** Total novel object visit time was significantly greater (*P* = 0.02) in the 8-week-old sow-reared group than other groups. Superscript letters (^*ab*^) denote differences (*P* < 0.05) between treatment means. AR; artificially reared pigs; PNW, postnatal week; SR, sow-reared pigs.

Overall, neither AR nor SR pigs at PNW 4 exhibited novelty preference (*P* > 0.05). At PNW 8, only SR pigs demonstrated a novelty preference, (*P* < 0.05) whereas AR pigs did not (*P* > 0.05; Figure 7). Pig exploratory behavior of all objects during the test trial of the novel object recognition task can be found in Table 6, while exploratory behavior of just the novel object and sample object is presented in Supplementary Tables 4, 5, respectively. While there was no rearing and age interaction effect observed for recognition index, SR pigs at PNW 8 exhibited higher recognition index compared with SR pigs at PNW 4, illustrating better recognition memory at PNW 8 (Figure 8). Age and interaction effects were observed for the total time spent exploring the novel object (*P* < 0.05; Supplementary Table 4). The SR pigs at PNW 8 spent more time (*P* < 0.05) exploring the novel object compared with other treatment groups. No other exploratory behavior measurements were dissimilar between the four treatment groups (*P* > 0.05).

**TABLE 6:** Exploratory behavior of all objects during the test trial of the NOR task^1^.

Measurements	RI	Total object visit time, s	Number of all object visits, n	Mean object visit time, s/visit	Latency to first object visit, s	Latency to last object visit, s
**Effect of rearing**						
AR	0.55	55.1	11.7	4.4	12.8	245.3
SR	0.55	75.8	13.9	5.0	7.7	273.0
SEM	0.050	10.45	1.14	0.73	4.00	13.98
**Effect of age**						
Week 4	0.47	58.9	12.1	4.2	11.3	254.2
Week 8	0.63	71.9	13.4	5.1	9.3	264.1
SEM	0.045	9.49	1.03	0.68	3.70	12.95
**Interaction means**						
AR:Week 4	0.52^ab^	58.3	11.7	4.4	13.9	235.0
AR:Week 8	0.57^ab^	51.9	11.6	4.3	11.7	255.6
SR:Week 4	0.42^a^	59.6	12.5	4.0	8.6	273.4
SR:Week 8	0.68^b^	92.0	15.3	5.9	6.8	272.7
SEM	0.074	15.74	1.73	1.07	5.76	20.06
***P*-value^2^**						
Rearing	0.949	0.102	0.105	0.475	0.261	0.081
Age	**0.010**	0.299	0.348	0.277	0.649	0.518
Rearing:Age	0.095	0.122	0.297	0.208	0.968	0.484

*^ab^Superscript letters denote differences between treatment means (P < 0.05).*

*^1^Pigs were reared in an artificial rearing system or with sows for the first 4 weeks of age. After the first 4 weeks of age, both groups were housed together in nursery pens. AR, artificially reared pigs; RI, recognition index; SEM, standard error of mean; SR, sow-reared pigs.*

*^2^P-values derived from repeated-measures ANOVA for the main effects and the interaction. Significant P-values are shown in bold text for emphasis.*

## Discussion

Artificially rearing pigs is a proven technique for investigations requiring precise control over environmental factors, including dietary interventions. However, scientific evidence is needed to determine the extent of influence exerted by early-life rearing environment on growth, behavior, and overall development of the pig. Therefore, we aimed to evaluate developmental patterns of brain macrostructure and microstructure, as well as functional capacity measured using recognition memory, to examine the influence of early-life rearing environment. The artificial rearing environment utilized in the current study involved individually housing pigs comfortably in custom rearing units with unlimited access to milk replacer and allowed pigs to see, hear, and smell neighboring pigs from PND 2 to PNW 4. Thus, resulting in a physical but not social isolation. Pigs that were reared by the sow remained in farrowing crates with their respective dam and littermates through PNW 4, per standard agricultural practices. Overall, no differences in longitudinal growth performance outcomes were noted between SR and AR pigs. The absolute volume developmental patterns for whole brain, GM, WM, and cerebrospinal fluid also did not differ due to early-life rearing environment and few differences in regional macrostructure were observed between the two groups. Maximum MWF did not differ between AR and SR pigs, but some regions exhibited higher overall rates of MWF development in AR than SR pigs. Furthermore, early-life rearing environment minimally influenced the patterns of FA development. Aside from SR pigs illustrating better recognition memory at PNW 8 than PNW 4, there were also minimal differences in behavioral outcomes between the two rearing groups.

### Brain Macrostructural Assessment

The pig is known to be one of the most appropriate regularly used preclinical models for human infants regarding brain development because its brain growth spurt, the period of growth when the brain is growing most rapidly, extends from the late prenatal to early postnatal period (Dobbing and Sands, 1979). Specifically, the most rapid period of brain growth (in terms of brain weight) for the pig was noted to occur from about 50 days before birth to about 40 days after birth (Dickerson and Dobbing, 1967). Comparably, the whole brain’s absolute volume for AR and SR pigs continually increased throughout the 24 week study period, but, in agreement with the aforementioned study, a rapid period of growth, indicated by a steep slope of the developmental curve, was present during the first 5 weeks of age (i.e., 35 days) (Figure 3A). Early-life rearing environment did not influence the development of major tissue types within the brain, as whole brain, GM, WM, and cerebrospinal fluid absolute volume developmental patterns did not differ between AR and SR pigs.

The maximum whole brain volume for the pig was estimated to be around 152,000 mm^3^ (Table 3), indicating a 3.5-fold increase in whole brain volume from the 44,000 mm^3^ volume at PNW 1 (Figure 5A). Accordingly, pig whole brain absolute volume at birth was around 28% of the adult volume. This coincides with conclusions from previous work that stated the brain weight of the pig at birth to be 25% of its adult brain weight (Dobbing and Sands, 1979). However, the current estimated maximum volume is larger than previous estimates of maximum whole brain volume of the pig (Conrad et al., 2012). The discrepancy in estimates is most likely due to the combination of utilizing different models to estimate the parameters, with a Gompertz model being applied in the current study and a logistic model utilized previously (Conrad et al., 2012) and from using an updated pig brain atlas in the current study (Fil et al., 2021). The estimated maximum volume of GM and WM was around 71,000 and 55,000 mm^3^, respectively (Table 3). Interestingly, GM is around three-fourths of the estimated maximum volume at PNW 24 (Figure 5B) while WM almost reaches its estimated maximum volume at PNW 24 (Figure 5C). This suggests that GM continues to increase through sexual maturity (i.e., approximately 24-weeks of age; Reiland, 1978), whereas WM is fully developed around sexual maturity in the pig.

Regional absolute volume developmental trajectories were similar between AR and SR pigs, with the exception of the left olfactory bulb and the left and right cortex that displayed a higher overall rate of development in SR pigs than AR pigs. Thus, indicating that the rate of reaching the mature volume of the left olfactory bulb and left and right cortex was faster in SR pigs than AR pigs. The faster rate of absolute volume development of the olfactory bulb may indicate more use of olfaction by SR pigs than AR pigs. In the Y-maze paradigm, pigs were found to be most attracted to the odors specific to their mother (i.e., skin secretions and maternal feces) while milk and colostrum were found to be neutral olfactory stimuli (Morrow-Tesch and McGlone, 1990b). Moreover, when pigs experienced olfactory deprivation by anesthetization of the olfactory epithelium, complete disruption of nipple attachment of baby pigs was observed (Morrow-Tesch and McGlone, 1990a). The SR pigs live in a competitive environment, where they must find and defend access to a preferential teat before a stable teat order is formed (De Passille et al., 1988). Thus, specific compounds produced by the sow modulate nipple attachment and may indicate which teats have been used most and therefore contain the most milk and nutrition. AR pigs had unlimited access to milk and may not have utilized the olfactory system as greatly as SR pigs, therefore resulting in a slower rate of absolute volume development of the left olfactory bulb. Although the rate of development differed between groups in the three regions, overall volume growth did not differ as the maximum absolute volumes of all regions were the same. Thus, higher growth rate did not necessarily influence absolute volume later in life.

### Brain Microstructural Assessment

Brain microstructure developmental patterns were assessed utilizing MWF and FA outcomes. The brain parenchyma has two micro-anatomical water pools, one that is associated with the water bound within the myelin sheath lipid bilayers and one that is associated with the water inside and outside the myelinated axon. MWF is the quantified myelin-bound water signal and its trends correlate to histological patterns of myelination (Deoni et al., 2011, 2012). Therefore, MWF is a surrogate measure of myelin and assists in providing insight into myelination patterns. Moreover, it most accurately represents myelin trends in areas with high volumes of white matter. Previous work by Pond et al. (2000) had evaluated myelin development in the pig by examining cholesterol accretion. They observed a curvilinear increase in cholesterol accretion from 70 to 140 days post-conception, with a rapid rise in velocity of cholesterol accretion throughout 120–140 days post-conception, or 1–3 weeks after birth (Pond et al., 2000). Likewise, Sweasey et al. (1976) observed biphasic myelination of the pig brain, where myelin was estimated by daily increases in tissue cerebroside concentrations. They observed myelination peaks at two occurrences, 2 weeks before birth and 3 weeks after birth (Sweasey et al., 1976). MWF developmental patterns for AR and SR pigs from the current study presented comparable outcomes, as there was a distinct increase in whole brain MWF from PNW 1–4, as is visually evident from the increased slope in Figure 6. The whole brain, along with several other ROI, exhibited higher rates of MWF development in AR pigs than SR pigs (Table 4). Indeed, AR pigs had a faster rate of MWF development than SR pigs in the cerebellum, left putamen and globus-pallidus, and midbrain, regions responsible for motor movement (Llinás and Welsh, 1993; Marchand et al., 2008; Hosp et al., 2011; Hegeman et al., 2016). Moreover, the left internal capsule, thalamus, and the pons, brain areas responsible for relaying sensory and motor signals (Basso et al., 2005; Brodal, 2014; Emos and Agarwal, 2020), had accelerated MWF development in AR pigs than SR pigs. Myelin formation can be regulated by activity to help adapt brain function to an environmental stimuli (Mount and Monje, 2017; Bechler et al., 2018). Thus, the increased rate of MWF development suggests that the observed regions were more active in the AR environment than the SR environment. A direct cause of the observed accelerated rates of MWF development cannot be determined, as early-life rearing environment differed in more than one factor including nutritional composition, *ad libitum* milk access through an automatic feeding system, and human-based care. However, we speculate that the faster maturation of myelin development in regions responsible for motor movement and relaying sensory and motor signals may be related to differences in oral feeding or management paradigms. The sucking reflex is an innate behavior expressed by mammals immediately after birth. However, AR pigs had to learn to drink milk from bowls rather than suckling on their mother staring on PND 2. Thus, AR pigs may have experienced differential development of specific brain regions to permit development of new ingestive behaviors. Although AR pigs exhibited an increased myelination rate during the first few weeks of life, these changes did not influence long-term development because no differences were observed between AR and SR pigs in terms of the plateau MWF parameter estimate.

Diffusion tensor imaging was performed to generate FA values as a measure for microstructural assessment. Similarly to what has been observed in domestic pigs previously, FA for all pigs and regions increased over time, spanning from 0.2 to 0.5 (Winter et al., 2011). The WM mask and FA mask estimates, which identified WM by using the WM probability map from the pig brain atlas and DTI-generated WM, respectively, were similar for AR and SR pigs. Thus, early-life rearing environment did not influence the overall microstructural developmental patterns of WM. AR and SR pigs exhibited similar developmental patterns of FA in all regions except a slightly higher rate of FA increase in the right internal capsule of SR pigs compared with AR pigs. These outcomes contrast a previous study performed by our laboratory that had observed higher average WM, left cortex, right cortex, and combined internal capsule FA values in SR pigs compared with AR pigs (Jacob et al., 2016). The previous cross-sectional study was performed to compare MRI outcomes on AR and SR pigs at PNW 3, while the current study examined development longitudinally from birth and through sexual maturity, which provides a more accurate representation of developmental changes. Thus, the FA outcomes from the current study suggest that early-life rearing environment might influence some orientation-dependent aspect of the microstructure in the right internal capsule that may be due to changes in axon diameter and/or density, membrane permeability, and/or myelination (Beaulieu, 2002; Jones et al., 2013). However, this difference in FA was not a lasting effect that could be observed into adulthood, as there were no differences observed in maximum FA values.

### Behavior

NOR is a well-established cognitive task that has been validated in several species including pigs (Fleming and Dilger, 2017) and measures recognition memory as an indicator of cognitive development. In the present study, the effects of early-life rearing environment on cognitive development was examined at twodifferent time-points using the NOR task. It is important to consider the relationship between the recognition index and the novel object exploration time in order to appropriately interpret the exploratory behavior outcomes. Recognition index is the proportion of time spent investigating the novel object compared to the total exploration time of both objects. Thus, the recognition index is highly correlated, but not equal to, the total time spent exploring the novel object.

SR pigs at PNW 8 exhibited more time spent investigating the novel object that translated into expression of novelty preference, which is demonstrated by a recognition index higher than the chance value of 0.5. Similar findings had been observed previously where older pigs exhibited higher novel object exploration time and greater exploratory behavior than younger pigs (Fleming and Dilger, 2017), which indicates that the NOR performance is age-dependent. Although only SR pigs in the current study exhibited increase in recognition memory, the age-dependent rearing effect on recognition memory was not observed. Most measures of exploratory behaviors such as latency to visit the object, number of object visits, and average time visiting objects were not influenced by early-life rearing environment. The relationship between latency and time spent visiting objects to anxiety-related exploratory behavior in pigs has been speculated previously (Joung et al., 2020), suggesting that rearing environment had no influence on anxiety-related behaviors. However, due to the limited NOR arena size, the current study was restricted to utilize pigs up to PNW 8. Therefore, further investigations should be conducted in pigs closer to the age of sexual maturity to better understand the long-term impact of early-life rearing environment on the onset of recognition memory improvement and exploratory behaviors.

Despite a lack of interaction effect between age and rearing system in recognition memory and minimal differences in exploratory behaviors, there are some factors that are inevitably different between artificial rearing and sow-rearing environment. One aspect of the sow-rearing environment is the constant opportunities to exhibit social play, especially between littermates. Peer play, especially during first weeks of a pig’s life, can be very important for behavioral and cognitive development (Telkänranta and Edwards, 2018). Early-life peer play can help develop a broad skill set including fine motor and social skills necessary for behavioral adaptability (Bekoff, 1984). In the current study, SR pigs had more opportunities to interact with peers including both littermates and non-littermates, while AR pigs inevitably had limited exposure to their peers by only having sensory but no physical access to neighbor pigs in their home-cage environment, and a daily direct and communal playtime of 15 min. Indeed, peer play in pigs begins about 3–5 days old and peaks at 2–6 weeks old, followed by a gradual decrease through sexual maturity (Newberry et al., 1988), and pigs that were repeatedly separated from the dam and littermates during the first 2 weeks of life displayed a significant decrease in locomotion and increase in inactivity compared with pigs that were not isolated (Kanitz et al., 2004). While these findings support that early-life peer play is important for motor, behavioral, and cognitive development, the early-life artificial rearing environment in the current study did not include complete social isolation. Although pigs were individually housed, they were in close proximity to one another (i.e., two pigs separated by a clear, perforated, plastic divider) and were allowed to see, smell, and hear adjacent conspecifics at all times. Moreover, the difference in the rearing system was only present during the first 4 weeks, as AR and SR pigs were reunited and reared in a group housing setting starting at PNW 4. In comparison, Kanitz and colleagues isolated pigs from PND 3 to 11 for 2 h each day (Kanitz et al., 2004). Therefore, it is challenging to directly compare the effects of social isolation in pigs due to the major differences in study design. Moreover, along with different types of peer interaction, AR pigs in our study experienced a different physical environment, *ad libitum* access to milk replacer (i.e., a different nutrient composition than porcine milk), and human-based care as compared with SR pigs. Thus, the differing behavioral outcomes between AR and SR pigs cannot be pinpointed to just one factor within the early-life rearing environments, yet differences in performance during the NOR task were minimal between early-life rearing groups.

### Limitations

Only intact male pigs were used for evaluating the influence of early-life rearing environment on development in the domestic pig. Over the age range of pigs studied here, both male and female pigs would be reproductively mature by study conclusion. As such, intact male pigs are both physically large and aggressive, which necessitates male and female pigs being raised separately. Therefore, one sex was utilized for this longitudinal study to reduce the variability in the dataset as it would have been necessary to raise the sexes in separate facilities and have different personnel manage those animals. Our neuroimaging methods and behavioral paradigm are not without their limitations as well. Higher variability of all MRI measures was observed at later time-points (PNW 18 and 24) than at the earlier times-points, which contributed to higher standard errors in certain brain regions. This relatively higher variability was attributed to the heavier and deeper breathing of larger pigs, which contributed to movement in the MRI scanner. Moreover, two different MRI coils and sequences had to be utilized to accommodate the massive differences in head size of pigs at PNW 1 vs. 24. Therefore, the spatial resolution differences in the imaging sequences are also a potential source of variability across the age groups. Further refinement of our sequences to provide higher resolution with sufficient signal-to-noise ratio will reduce this variability in future investigations. Furthermore, the NOR task successfully measures recognition memory during early-life in pigs, however, the addition of a wider array of behavioral tests investigating other measures of cognitive development, such as radial arm maze or social recognition task, was not plausible due to the restrictions in the number of pigs and the timeline of the study design. Therefore, it is important to emphasize that the minimal differences found between AR and SR pigs are strictly for the object recognition memory and exploratory behaviors driven from NOR task, and the results should not be generalized to overall cognitive and behavioral development.

## Conclusion

This study provided longitudinal, normative brain developmental patterns for the domestic pig and evaluated whether early-life rearing environment influenced neurodevelopmental and behavioral outcomes. Overall, early-life rearing environment had no influence on growth performance or general health of pigs. Only minor differences in brain developmental trajectories were observed for absolute brain volume and FA outcomes. AR pigs experienced higher rates of myelination in motor and coordination-related regions compared with SR pigs, but there was no evidence that this caused permanent alterations in brain structure or function. No overall differences in recognition memory were observed between AR pigs and SR pigs, except SR pigs at PNW 8 demonstrating higher recognition index, but the differences are little given that performance on NOR task is age-dependent. Moreover, there were minimal differences in general exploratory behavioral measurements between the treatment groups. This study provides strong evidence that artificially rearing pigs results in minimal differences in brain development and object recognition memory than sow-rearing.

## Data Availability Statement

The raw data supporting the conclusions of this article will be made available by the authors, without undue reservation.

## Ethics Statement

The animal study was reviewed and approved by the Institutional Animal Care and Use Committee, University of Illinois, Urbana, IL.

## Author Contributions

JF, SJ, CH, and RD contributed to the design of the study, interpreted the study, and prepared the manuscript. All authors were involved in data acquisition, analysis, and interpretation, read and approved the final version of this manuscript.

## Conflict of Interest

RD was consulted for and received grant funding from Nestlé. The remaining authors declare that the research was conducted in the absence of any commercial or financial relationships that could be construed as a potential conflict of interest.

## Abbreviations

AR: Artificially reared
BIC: Bayesian information criterion
BW: body weight
DTI: diffusion tensor imaging
TE: echo time
FA: fractional anisotropy
GM: gray matter
MRI: magnetic resonance imaging
MWF: myelin water fraction
NOR: novel object recognition
PND: postnatal day
PNW: postnatal week
TR: repetition time
SR: sow-reared
SSFP: steady-state free precession
WM: white matter.

## References

[B1] Basso M. A. Uhlrich D. Bickford M. E. (2005). Cortical function: a view from the thalamus. *Neuron* 45 485–488. 10.1016/j.neuron.2005.01.035 15756758

[B2] Baxter E. M. Rutherford K. M. D. D’Eath R. B. Arnott G. Turner S. P. Sandøe P. (2013). The welfare implications of large litter size in the domestic pig II: management factors. *Anim. Welf.* 22 219–238. 10.7120/09627286.22.2.219

[B3] Beaulieu C. (2002). The basis of anisotropic water diffusion in the nervous system – A technical review. *NMR Biomed.* 15 435–455. 10.1002/nbm.782 12489094

[B4] Bechler M. E. Swire M. Ffrench-Constant C. (2018). Intrinsic and adaptive myelination—A sequential mechanism for smart wiring in the brain. *Dev. Neurobiol.* 78 68–79. 10.1002/dneu.22518 28834358 PMC5813148

[B5] Bekoff M. (1984). Social play behavior. *Bioscience* 34 228–233. 10.2307/1309460

[B6] Bleasdale J. K. A. Nelder J. A. (1960). Plant population and crop yield. *Nature* 188:342.

[B7] Braude R. Mitchell K. G. Newport M. J. Porter J. W. G. (1983). Artificial rearing of pigs: 1. Effect of frequency and level of feeding on performance and digestion of milk proteins. *Br. J. Nutr.* 24 501–516. 10.1079/BJN19700049 4318116

[B8] Brodal P. (2014). Pons. *Encycl. Neurol. Sci.* 3 936–937. 10.1016/B978-0-12-385157-4.01172-6

[B9] Cabrera R. A. Boyd R. D. Jungst S. B. Wilson E. R. Johnston M. E. Vignes J. L. (2010). Impact of lactation length and piglet weaning weight on long-term growth and viability of progeny. *J. Anim. Sci.* 88 2265–2276. 10.2527/jas.2009-2121 20190163

[B10] Conrad M. S. Dilger R. N. Johnson R. W. (2012). Brain growth of the domestic pig (*sus scrofa*) from 2 to 24 weeks of age: a longitudinal MRI study. *Dev. Neurosci.* 34 291–298. 10.1159/000339311 22777003 PMC3646377

[B11] De Passille A. M. B. Rushen J. Hartsock T. G. (1988). Ontogeny of teat fidelity in pigs and its relation to competition at suckling. *Can. J. Anim. Sci.* 68 325–338. 10.4141/cjas88-037

[B12] Dean D. C. III O’Muircheartaigh J. Dirks H. Waskiewicz N. Lehman K. Walker L. (2014). Modeling healthy male white matter and myelin development: 3 through 60 months of age. *Neuroimage* 84 742–752. 10.1016/j.neuroimage.2013.09.058 24095814 PMC3895775

[B13] Deoni S. C. L. Dean D. C. O’Muircheartaigh J. Dirks H. Jerskey B. A. (2012). Investigating white matter development in infancy and early childhood using myelin water faction and relaxation time mapping. *Neuroimage* 63 1038–1053. 10.1016/j.neuroimage.2012.07.037 22884937 PMC3711836

[B14] Deoni S. C. L. Mercure E. Blasi A. Gasston D. Thomson A. Johnson M. (2011). Mapping infant brain myelination with magnetic resonance imaging. *J. Neurosci.* 31 784–791. 10.1523/JNEUROSCI.2106-10.2011 21228187 PMC6623428

[B15] Dickerson J. W. T. Dobbing J. (1967). Prenatal and postnatal growth and development of the central nervous system of the pig. *Proc. R. Soc. Lond. Ser. B. Biol. Sci.* 166 384–395. 10.1086/303379 24796035

[B16] Dobbing J. Sands J. (1979). Comparative aspects of the brain growth spurt. *Early Hum. Dev.* 311 79–83. 10.1016/0378-3782(79)90022-7 118862

[B17] Emos M. C. Agarwal S. (2020). “Neuroanatomy, internal capsule,” in *StatPearls [Internet]*, (Treaure Island, FL: StatPearls Publishing). Available online at: https://www.ncbi.nlm.nih.gov/books/NBK542181/ (accessed Aug 10, 2020). 31194338

[B18] Fil J. E. Fleming S. A. Chichlowski M. Gross G. Berg B. M. Dilger R. N. (2019). Evaluation of dietary bovine milk fat globule membrane supplementation on growth, serum cholesterol and lipoproteins, and neurodevelopment in the young pig. *Front. Pediatr.* 7:417. 10.3389/fped.2019.00417 31681715 PMC6811645

[B19] Fil J. E. Joung S. Zimmerman B. J. Sutton B. P. Dilger R. N. (2021). High-resolution magnetic resonance imaging-based atlases for the young and adolescent domesticated pig (*Sus scrofa*). *J. Neurosci. Methods* 354:109107. 10.1016/j.jneumeth.2021.109107 33675840

[B20] Fleming S. A. Dilger R. N. (2017). Young pigs exhibit differential exploratory behavior during novelty preference tasks in response to age, sex, and delay. *Behav. Brain Res.* 321 50–60. 10.1016/j.bbr.2016.12.027 28042005

[B21] Gompertz B. (1825). On the nature of the function expressive of the law of human mortality, and on a new mode of determining the value of life contingencies. *Philos. Trans. R. Soc. Lond. Ser. B. Biol. Sci.* 115 513–583. 10.1098/rstl.1825.0026 PMC4360127 25750242

[B22] Hegeman D. J. Hong E. S. Hernández V. M. Chan C. S. (2016). The external globus pallidus: progress and perspectives. *Eur. J. Neurosci.* 43 1239–1265. 10.1111/ejn.13196 26841063 PMC4874844

[B23] Henare S. J. Mellor D. J. Lentle R. G. Moughan P. J. (2008). An appraisal of the strengths and weaknesses of newborn and juvenile rat models for researching gastrointestinal development. *Lab. Anim.* 42 231–245. 10.1258/la.2007.007034 18625579

[B24] Hosp J. A. Pekanovic A. Rioult-Pedotti M. S. Luft A. R. (2011). Dopaminergic projections from midbrain to primary motor cortex mediate motor skill learning. *J. Neurosci.* 31 2481–2487. 10.1523/JNEUROSCI.5411-10.2011 21325515 PMC6623715

[B25] Jacob R. M. Mudd A. T. Alexander L. S. Lai C.-S. Dilger R. N. (2016). Comparison of brain development in sow-reared and artificially reared piglets. *Front. Pediatr.* 4:95. 10.3389/fped.2016.00095 27672632 PMC5018487

[B26] Jenkinson M. Beckmann C. F. Behrens T. E. J. Woolrich M. W. Smith S. M. (2012). FSL. *Neuroimage* 62 782–790. 10.1016/j.neuroimage.2011.09.015 21979382

[B27] Jones D. K. Knösche T. R. Turner R. (2013). White matter integrity, fiber count, and other fallacies: the do’s and don’ts of diffusion MRI. *Neuroimage* 73 239–254. 10.1016/j.neuroimage.2012.06.081 22846632

[B28] Joung S. Fil J. E. Heckmann A. B. Kvistgaard A. S. Dilger R. N. (2020). Early-life supplementation of bovine milk osteopontin supports neurodevelopment and influences exploratory behavior. *Nutrients* 12:2206. 10.3390/nu12082206 32722080 PMC7469054

[B29] Kanitz E. Tuchscherer M. Puppe B. Tuchscherer A. Stabenow B. (2004). Consequences of repeated early isolation in domestic piglets (*Sus scrofa*) on their behavioural, neuroendocrine, and immunological responses. *Brain Behav. Immun.* 18 35–45. 10.1016/S0889-1591(03)00085-0 14651945

[B30] Kinder H. A. Baker E. W. West F. D. (2019). The pig as a preclinical traumatic brain injury model: current models, functional outcome measures, and translational detection strategies. *Neural Regen. Res.* 14 413–424. 10.4103/1673-5374.245334 30539807 PMC6334610

[B31] Lind N. M. Moustgaard A. Jelsing J. Vajta G. Cumming P. Hansen A. K. (2007). The use of pigs in neuroscience: modeling brain disorders. *Neurosci. Biobehav. Rev.* 31 728–751. 10.1016/j.neubiorev.2007.02.003 17445892

[B32] Llinás R. Welsh J. P. (1993). On the cerebellum and motor learning. *Curr. Opin. Neurobiol.* 3 958–965. 10.1016/0959-4388(93)90168-X 8124080

[B33] Marchand W. R. Lee J. N. Thatcher J. W. Hsu E. W. Rashkin E. Suchy Y. (2008). Putamen coactivation during motor task execution. *Neuroreport* 19 957–960. 10.1097/WNR.0b013e328302c873 18521000

[B34] Morrow-Tesch J. McGlone J. J. (1990a). Sensory systems and nipple attachment behavior in neonatal pigs. *Physiol. Behav.* 47 1–4. 10.1016/0031-9384(90)90034-2 2326324

[B35] Morrow-Tesch J. McGlone J. J. (1990b). Sources of maternal odors and the development of odor preferences in baby pigs. *J. Anim. Sci.* 68 3563–3571. 10.2527/1990.68113563x 2262409

[B36] Mount C. W. Monje M. (2017). Wrapped to adapt: experience-dependent myelination. *Neuron* 95 743–756. 10.1016/j.neuron.2017.07.009 28817797 PMC5667660

[B37] Mudd A. T. Dilger R. N. (2017). Early-life nutrition and neurodevelopment: use of the piglet as a translational model. *Adv. Nutr.* 8 92–104. 10.3945/an.116.013243 28096130 PMC5227977

[B38] Mudd A. T. Fil J. E. Knight L. C. Lam F. Liang Z.-P. Dilger R. N. (2018). Early-life iron deficiency reduces brain iron content and alters brain tissue composition despite iron repletion: a neuroimaging assessment. *Nutrients* 10:135. 10.3390/nu10020135 29382055 PMC5852711

[B39] National Research Council (2012). *Nutrient Requirements of Swine.* Washington, DC: National Academies Press.

[B40] Newberry R. Wood-Gush D. G. Hall J. W. (1988). Playful behaviour of piglets. *Behav. Process.* 17 205–216. 10.1016/0376-6357(88)90004-6 24897547

[B41] Pond W. G. Boleman S. L. Fiorotto M. L. Ho H. Knabe D. A. Mersmann H. J. (2000). Perinatal ontogeny of brain growth in the domestic pig. *Proc. Soc. Exp. Biol. Med.* 223 102–108. 10.1111/j.1525-1373.2000.22314.x 10632968

[B42] Reiland S. (1978). Growth and skeletal development of the pig. *Acta Radiol. Suppl.* 358 15–22. 233594

[B43] Richards F. J. (1959). A flexible growth function for empirical use. *J. Exp. Bot.* 10 290–301. 10.1093/jxb/10.2.290 12432039

[B44] Rzezniczek M. Gygax L. Wechsler B. Weber R. (2015). Comparison of the behaviour of piglets raised in an artificial rearing system or reared by the sow. *Appl. Anim. Behav. Sci.* 165 57–65. 10.1016/j.applanim.2015.01.009

[B45] Saikali S. Meurice P. Sauleau P. Eliat P.-A. Bellaud P. Randuineau G. (2010). A three-dimensional digital segmented and deformable brain atlas of the domestic pig. *J. Neurosci. Methods* 192 102–109. 10.1016/j.jneumeth.2010.07.041 20692291

[B46] Schmitt O. O’Driscoll K. Boyle L. A. Baxter E. M. (2019). Artificial rearing affects piglets pre-weaning behaviour, welfare and growth performance. *Appl. Anim. Behav. Sci.* 210 16–25. 10.1016/j.applanim.2018.10.018

[B47] Schwarz G. (1978). Estimating the dimension of a model. *Ann. Stat.* 6 461–464.

[B48] Sciascia Q. Das G. Metges C. C. (2016). Review: the pig as a model for humans: effects of nutritional factors on intestinal function and health. *Am. Soc. Anim. Sci.* 94 441–452. 10.2527/jas.2015-9788 32704858

[B49] Stannard C. J. Williams A. P. Gibbs P. A. (1985). Temperature/growth relationships for psychrotrophic food-spoilage bacteria. *Food Microbiol.* 2 115–122. 10.1016/S0740-0020(85)80004-6

[B50] Sweasey D. Patterson D. S. P. Glancy E. M. (1976). Biphasic myelination and the fatty acid composition of cerebrosides and cholesterol esters in the developing central nervous system of the domestic pig. *J. Neurochem.* 27 375–380. 10.1111/j.1471-4159.1976.tb12256.x 965978

[B51] Swindle M. M. Smith A. C. Laber-Laird K. Dungan L. (1994). Swine in biomedical research: management and models. *ILAR J.* 36 234–241. 10.1093/ilar.36.1.1

[B52] Telkänranta H. Edwards S. A. (2018). “Lifetime consequences of the early physical and social environment of piglets,” in *Advances in Pig Welfare. (Woodhead Publishing series in food science, technology and nutrition)*, 1 Edn, ed. Spinka M. (Amsterdam: Elsevier), 101–136. 10.1016/B978-0-08-101012-9.00013-7

[B53] Wagner K. R. Xi G. Hua Y. Zuccarello M. De Courten-Myers G. M. Broderick J. P. (1999). Ultra-early clot aspiration after lysis with tissue plasminogen activator in a porcine model of intracerebral hemorrhage: edema reduction and blood-brain barrier protection. *J. Neurosurg.* 90 491–498. 10.3171/jns.1999.90.3.0491 10067918

[B54] Winter J. D. Dorner S. Lukovic J. Fisher J. A. St. Lawrence K. S. Kassner A. (2011). Noninvasive MRI measures of microstructural and cerebrovascular changes during normal swine brain development. *Pediatr. Res.* 69 418–424. 10.1203/PDR.0b013e3182110f7e 21258264

[B55] Yasuda H. Harauma A. Kato M. Ootomo Y. Hatanaka E. Moriguchi T. (2016). Artificially reared mice exhibit anxiety-like behavior in adulthood. *Exp. Anim.* 65 267–274. 10.1538/expanim.15-0115 26948536 PMC4976240

