# Supplementary material for: Influence of Rearing Environment on Longitudinal Brain Development, Object Recognition Memory, and Exploratory Behaviors in the Domestic Pig (Sus scrofa)
Source: Front Neurosci. 2021 Mar 24;15:649536. doi: 10.3389/fnins.2021.649536 (PMC8024486; doi:10.3389/fnins.2021.649536)
Supplement: Supplementary file 1 [file Table_1.docx]

| **Supplementary Table 1.** Bayesian information criterion and rank sum values for sigmoid-type models from regional absolute volumes^1^ | | | | | | | | | |
| --- | --- | --- | --- | --- | --- | --- | --- | --- | --- |
| **ROI** | **Gompertz** | **Logistic^2^** | **Logistic^3^** | **Modified Gompertz** | **Generalized Logistic** | **Bleasdale and Nelder** | **Hyperbolic Tangent** | **Richards** | **Stannard** |
| Cerebral aqueduct | 1808.929 | **1778.376** | 559166.9 | 297100.8 | 1814.728 | 309529.7 | 50922.83 | 1791.316 | 829252.9 |
| Corpus callosum | **3386.81** | 16430668 | 3793.249 | 67488360 | 2476246 | 1.11E+08 | 3798.752 | 10583744 | 42405951 |
| Cerebellum | **5889.466** |  |  |  | 3.94E+09 |  | 3.09E+11 | 3.06E+11 |  |
| Fourth ventricle | **1694.612** | 3940.199 | 2432.554 | 2472.434 | 3090.409 | 2739907 | 385107.8 | 2372.622 | 2341562 |
| Hypothalamus | 6994047 | 1680207 | **3456.445** | 38648406 | 20603518 | 53407819 | 3795.156 | 9393723 | 25416777 |
| Left caudate | **3274.198** | 4064745 | 3792.917 | 70160153 | 2602996 | 1.23E+08 | 3810.595 | 1773548 | 43480275 |
| Left hippocampus |  | 4504.011 | **4304.51** | 2.95E+08 | 4364.393 | 5.11E+08 | 4358.825 | 2.62E+08 | 3.26E+08 |
| Left inferior colliculus | 5031.634 | **3907.043** | 4547.978 | 4.46E+08 | 33515194 | 8.08E+08 | 6.20E+08 | 5.94E+08 | 3.75E+08 |
| Left internal capsule | 410408.2 | 558484.1 | 52591688 | 11020459 | 3523010 | 18662099 | **8814.593** | 10408600 | 10276479 |
| Left olfactory bulb | 5680.26 | 5677.203 | **5492.868** | 1.11E+10 | 5110992 |  | 5.49E+08 | 3.61E+09 | 1.09E+10 |
| Left putamen-globus pallidus | 7656631 | 2779331 | **3318.675** | 18765812 | 9426246 | 67554511 | 32270.98 | 8407537 | 9436363 |
| Left superior colliculus | 3981.736 | 4100.159 | **3852.793** | 1.26E+08 | 96963292 | 1.69E+08 | 3960.822 | 3979673 | 87915113 |
| Lateral ventricle | 4272.397 | **3667.186** | 4063.797 | 1.16E+08 | 1.94E+08 | 2.99E+08 | 1.43E+08 | 4069.267 | 1.01E+08 |
| Medulla | 5741.039 | 5795.577 | **5696.179** | 3.56E+09 |  | 2.38E+10 | 2.67E+10 | 2.14E+10 | 2.14E+10 |
| Midbrain | 4882.604 | **4520.076** | 5765.854 | 3.20E+09 | 3.12E+09 | 9.44E+08 | 2.32E+10 | 2.81E+10 | 2.42E+09 |
| Pons | 4692.592 | 4734.227 | 4**378.309** | 9.04E+09 |  | 12278.67 | 1.24E+10 | 1.19E+10 | 9.89E+09 |
| Right caudate | **3224.194** | 2780010 | 11714302 | 66986597 | 9742538 | 1.10E+08 | 7856244 | 3797.461 | 41651416 |
| Right hippocampus | 3593.952 | **3187.845** | 3623.085 | 3.63E+08 | 11449956 | 5.01E+08 | 4.57E+08 | 4427.238 | 2.82E+08 |
| Right inferior colliculus | 5057.698 | 4579.152 | **4480.169** | 4.43E+08 | 9.65E+08 | 7.94E+08 | 6.02E+08 | 5.87E+08 | 3.49E+08 |
| Right internal capsule | **2315721** | 65183423 | 58435371 | 10788526 | 3067115 | 19272552 | 7924172 | 8827354 | 8939085 |
| Right olfactory bulb | 5060.477 | **4990.57** | 5405.102 | 7.62E+09 |  | 1.41E+09 | 5406.375 | 5459.064 | 2.78E+08 |
| Right putamen-globus pallidus | **2752.494** | 1788144 | 2142463 | 8971218 | 7176124 | 51738338 | 3178.279 | 17176283 | 7158513 |
| Right superior colliculus | 3842.108 | **2877.068** | 3838.412 | 1.20E+08 | 97116995 | 1.13E+08 | 271941.2 | 3850.9 | 81978830 |
| Thalamus | **4264.82** | 5393.086 | 5062.406 | 2.28E+09 | 1.78E+09 |  | 1.99E+09 | 3.50E+09 | 2.35E+09 |
| ***Rank Sum*** | ***170*** | *154* | *157* | *69* | *96* | *39* | *116* | *108* | *81* |
| ^1^Bolded values indicate the lowest Bayesian information criterion for that particular region of interest  ^2^Logistic model used by Dean et al., 2014.  ^3^Logistic model used by Conrad et al., 2012. | | | | | | | | | |

| **Supplementary Table 2.** Bayesian information criterion and rank sum values for sigmoid-type models from myelin water fraction^1^ | | | | | | | | | |
| --- | --- | --- | --- | --- | --- | --- | --- | --- | --- |
| **ROI** | **Gompertz** | **Logistic^2^** | **Logistic^3^** | **Modified Gompertz** | **Generalized Logistic** | **Bleasdale and Nelder** | **Hyperbolic Tangent** | **Richards** | **Stannard** |
| Corpus callosum | -2366.53 | 7855.991 | 31217.05 | -2082.75 | -2074.95 | -2073 | -2081.66 | **-2562.14** | -2082.47 |
| Cerebellum | -2600.21 | 77716.32 | **-2600.6** | -2522.61 | -2554.44 | -2562.14 | -2562.14 | -2175.6 | -2562.14 |
| Combined cortex | **-2761.92** | 48891.29 | 26675.2 | -2409.1 | -2399.73 | -2408.86 | -2395.76 | -1904.01 | -2398.93 |
| Combined hippocampus | -2135 | 143231.6 | 29021.53 | -2179.98 | -2175.49 | -2180.82 | -2173.93 | **-2218.27** | -2170.07 |
| Combined internal capsule | **-2340.79** | 23081.85 | 37884.48 | -1903.45 | -1896.38 | -1899.98 | -1903.63 | -2309.17 | -1903.52 |
| Hypothalamus | **-2546.74** | 72500.06 | 29199.85 | -2218.32 | -2210.62 | -2213.61 | -2218.28 | -2459.34 | -2218.29 |
| Left cortex | **-2584.11** | 48749.77 | 26766.2 | -1999.36 | -2305.79 | -2291.7 | -2310.77 | -2061.74 | -2312.7 |
| Left hemisphere | -2222.01 | 70853.97 | 28347.39 | **-2459.72** | -2451.71 | -2452.87 | -2445.14 | -2170.32 | -2459.65 |
| Left hippocampus | **-2178.43** | 59074.51 | 29297.04 | -2063.24 | -2055.76 | -2060.08 | -2063 | -1825.14 | -2061.16 |
| Left inferior colliculus | -1987.45 | 10088.59 | 22205.04 | -2170.32 | -2162.62 | -2170.32 | -2170.32 | **-2217.34** | -2170.32 |
| Left internal capsule | **-2225.03** | 22459.6 | 39320.81 | -1825.22 | -1817.75 | -1821.98 | -1825.15 | -1641.57 | -1825.43 |
| Left olfactory bulb | -2185.42 | 30818.41 | 8917.216 | -2217.34 | -2209.64 | **-2217.34** | -2217.34 | -2177.97 | -2217.34 |
| Left putamen-globus pallidus | **-2317.95** | 103720.5 | 22078.74 | -1916.74 | -1887.56 | -1917.43 | -1916.74 | -2242.28 | -1915.45 |
| Left superior colliculus | **-2676.55** | 107326.5 | 21296.44 | -2177.75 | -2170.56 | -2176.85 | -2172.76 | -2519.04 | -2177.98 |
| Medulla | -2408.97 | 9181.502 | 21664.65 | -2242.28 | -2234.58 | -2242.28 | -2242.28 | **-2434.59** | -2242.28 |
| Midbrain | **-2747.59** | 115433.8 | 22006.57 | -2520.5 | -2514.53 | -2501.22 | -2516.7 | -2404.2 | -2521.89 |
| Pons | **-2789.38** | 13402.46 | 27468.25 | -2434.59 | -2426.89 | -2434.59 | -2427.74 | -2580.32 | -2434.59 |
| Right cortex | **-2568.65** | 136909.5 | 26589.06 | -2397.53 | -2396.2 | -2402.63 | -2380.98 | -2165.63 | -2403.54 |
| Right hemisphere | -2204.57 | 61815.1 | 27347.11 | **-2585.73** | -2578.45 | -2563.09 | -2556.55 | -2191.68 | -2585.19 |
| Right hippocampus | -2114.64 | 142679.1 | 28951.29 | **-2168.1** | -2160.68 | -2147.4 | -2152.77 | -1902 | -2163.65 |
| Right inferior colliculus | -2175.06 | 8944.044 | 20138.18 | -2191.87 | -2184.14 | -2188.38 | -2191.75 | -2167.37 | **-2191.84** |
| Right internal capsule | **-2286.84** | 23087.04 | 38722.7 | -1902.17 | -1895.55 | -1896.07 | -1903.09 | -2059.28 | -1903.03 |
| Right olfactory bulb | **-2389.32** | -2188.56 | 8290.125 | -2167.37 | -2159.67 | -2167.37 | -2167.37 | -2161.37 | -2167.37 |
| Right putamen-globus pallidus | **-2360.25** | 370.3224 | 35039.93 | -2060.08 | -2053.06 | -2060.32 | -2059.82 | -1961.62 | -2060.41 |
| Right superior colliculus | **-2776.99** | 94260.66 | 34392.76 | -2161.54 | -2153.57 | -2158.51 | -2160.14 | -2659.26 | -2161.5 |
| Thalamus | -2366.53 | 129595.6 | 26972.25 | -1972.79 | -1936.86 | -1965.76 | -1976.76 | **-2562.14** | -1977.57 |
| Whole brain | -2600.21 | 73812.49 | 28238.94 | **-2659.26** | -2651.56 | -2643 | -2639.39 | -2175.6 | -2647.59 |
| ***Rank Sum*** | ***197*** | *43* | *51* | *171* | *119* | *142* | *144* | *146* | *171* |
| ^1^Bolded values indicate the lowest Bayesian information criterion for that particular region of interest  ^2^Logistic model used by Dean et al., 2014.  ^3^Logistic model used by Conrad et al., 2012. | | | | | | | | | |

| **Supplementary Table 3.** Bayesian information criterion and rank sum values for sigmoid-type models from fractional anisotropy values^1^ | | | | | | | | | | |
| --- | --- | --- | --- | --- | --- | --- | --- | --- | --- | --- |
| **ROI^1^** | **Gompertz** | **Logistic^2^** | **Logistic^3^** | **Modified Gompertz** | **Generalized Logistic** | **Bleasdale and Nelder** | **Hyperbolic Tangent** | **Richards** | **Stannard** |  |
| Corpus callosum | **-1705.04** | -983.947 | -842.018 | -1223.3 | -1181.45 | -1191.56 | -1233.6 | -1195.99 | -1223.78 |  |
| Cerebellum | -1663.98 | -941.485 | **-1724.72** | -1643.08 | -1602.85 | -1643.08 | -1643.08 | -1643.08 | -1643.08 |  |
| Left caudate | -1606.83 | -1659.5 | **-1663.33** | -1526.73 | -1519.07 | -1526.73 | -1526.73 | -1526.73 | -1487.73 |  |
| Left hippocampus | -1622.76 | -1679.78 | **-1698.74** | -1617.03 | -1609.37 | -1572.36 | -1617.03 | -1604.72 | -1617.03 |  |
| Left internal capsule | -1112.19 | -887.512 | -1559.6 | **-1648.48** | -1603.27 | -1646 | -1610.59 | -1580.74 | -1648.46 |  |
| Left side | **-1758.12** | -1119.91 | -994.425 | -1086.99 | -1040.4 | -1049.39 | -1057.31 | -1098.15 | -1091.26 |  |
| Right caudate | -1124.6 | -1380.47 | **-1757.5** | -1306.43 | -1312.31 | -1288.47 | -1335.66 | -1348.87 | -1321.88 |  |
| Right hippocampus | **-1589.24** | -1522.25 | -1517.24 | -1484.17 | -1476.51 | -1484.17 | -1484.17 | -1440.68 | -1436.86 |  |
| Right internal capsule | -1370.47 | **-1683.92** | -1000.32 | -1610.84 | -1575.71 | -1610.84 | -1610.84 | -1552.82 | -1610.84 |  |
| Right side | -1671.58 | -1066.44 | **-1690.42** | -1081.32 | -1070.03 | -1045.84 | -1106.86 | -1102.69 | -1086.55 |  |
| Thalamus | **-1705.04** | -1578.39 | -1541.79 | -1561.03 | -1504.22 | -1526.02 | -1523.48 | -1504.17 | -1561.03 |  |
| FA Mask | **-1663.98** | -959.388 | -1569.08 | -1291.63 | -1293.26 | -1322.59 | -1308.96 | -1262.15 | -1280.02 |  |
| White matter Mask | -1606.83 | **-1668.13** | -842.018 | -1621.8 | -1553.12 | -1621.8 | -1621.8 | -1587.02 | -1621.8 |  |
| ***Rank Sum*** | ***74*** | *73* | *72* | *61* | *40* | *49* | *64* | *48* | *59* |  |
| ^1^Bolded values indicate the lowest Bayesian information criterion for that particular region of interest  ^2^Logistic model used by Dean et al., 2014.  ^3^Logistic model used by Conrad et al., 2012. | | | | | | | | | | |

| **Supplementary Table 4.** Exploratory behavior of the novel object during the test trial of the NOR task^1^ | | | | | |
| --- | --- | --- | --- | --- | --- |
| **Measurements** | **Total novel object visit time, s** | **Number of novel object visits, n** | **Mean novel object visit time, s/visit** | **Latency to first novel object visit, s** | **Latency to last novel object visit, s** |
| *Effect of rearing* |  |  |  |  |  |
| AR | 30.3 | 5.8 | 4.7 | 28.7 | 213.3 |
| SR | 45.8 | 7.3 | 5.6 | 21.5 | 226.4 |
| SEM | 7.74 | 0.63 | 0.98 | 9.16 | 19.75 |
| *Effect of age* |  |  |  |  |  |
| Week 4 | 28.0 | 5.9 | 4.3 | 25.5 | 204.0 |
| Week 8 | 48.2 | 7.2 | 6.0 | 24.8 | 235.8 |
| SEM | 7.14 | 0.57 | 0.91 | 8.48 | 18.38 |
| *Interaction means* |  |  |  |  |  |
| AR:Week 4 | 29.7^a^ | 5.7 | 4.6 | 25.1 | 197.9 |
| AR:Week 8 | 30.9^a^ | 5.8 | 4.8 | 32.4 | 228.8 |
| SR:Week 4 | 26.2^a^ | 6.1 | 3.9 | 25.9 | 210.0 |
| SR:Week 8 | 65.5^b^ | 8.5 | 7.2 | 17.1 | 242.8 |
| SEM | 10.94 | 0.96 | 1.39 | 13.21 | 27.74 |
| *P-value^2^* |  |  |  |  |  |
| Rearing | 0.067 | 0.057 | 0.427 | 0.487 | 0.541 |
| Age | **0.015** | 0.104 | 0.098 | 0.944 | 0.128 |
| Rearing:Age | **0.020** | 0.141 | 0.142 | 0.421 | 0.965 |
| ^ab^Superscript letters denote differences between treatment means (*P* < 0.05). | | | | | |
| ^1^Pigs were reared in an artificial rearing system or with sows for the first 4 weeks of age. After the first 4 weeks of age, both groups were housed together in nursery pens. Abbreviation: AR, artificially reared pigs; SEM, standard error of mean; SR, sow-reared pigs. | | | | | |
| ^2^ P-values derived from repeated-measures ANOVA for the main effects and the interaction. Significant P-values are shown in bold text for emphasis. | | | | | |

| **Supplementary Table 5.** Exploratory behavior of the sample object during the test trial of the NOR task^1^ | | | | | |
| --- | --- | --- | --- | --- | --- |
| **Measurements** | **Total sample object visit time, s** | **Number of sample object visits, n** | **Mean sample object visit time, s/visit** | **Latency to first sample object visit, s** | **Latency to last sample object visit, s** |
| *Effect of rearing* |  |  |  |  |  |
| AR | 25.0 | 5.9 | 3.7 | 25.8 | 219.3 |
| SR | 29.3 | 6.6 | 4.3 | 25.4 | 247.4 |
| SEM | 5.68 | 0.78 | 0.65 | 6.89 | 18.23 |
| *Effect of age* |  |  |  |  |  |
| Week 4 | 30.6 | 6.2 | 4.1 | 26.5 | 232.0 |
| Week 8 | 23.7 | 6.3 | 3.9 | 24.7 | 234.7 |
| SEM | 5.15 | 0.71 | 0.59 | 6.32 | 16.99 |
| *Interaction means* |  |  |  |  |  |
| AR:Week 4 | 28.9 | 6.0 | 4.0 | 23.6 | 217.6 |
| AR:Week 8 | 21.1 | 5.7 | 3.5 | 27.9 | 221.1 |
| SR:Week 4 | 32.2 | 6.5 | 4.2 | 29.4 | 246.4 |
| SR:Week 8 | 26.3 | 6.8 | 4.3 | 21.5 | 248.4 |
| SEM | 8.63 | 1.19 | 0.98 | 10.46 | 25.47 |
| *P-value^2^* |  |  |  |  |  |
| Rearing | 0.536 | 0.413 | 0.500 | 0.967 | 0.155 |
| Age | 0.317 | 0.965 | 0.807 | 0.828 | 0.887 |
| Rearing:Age | 0.889 | 0.743 | 0.708 | 0.470 | 0.966 |
| ^1^Pigs were reared in an artificial rearing system or with sows for the first 4 weeks of age. After the first 4 weeks of age, both groups were housed together in nursery pens. Abbreviation: AR, artificially reared pigs; NOR, novel object recognition; SEM, standard error of mean; SR, sow-reared pigs. | | | | | |
| ^2^ P-values derived from repeated-measures ANOVA for the main effects and the interaction. | | | | | |
